# Serosurvey and Risk Factors Associated with *Brucella* Infection in High Risk Occupations from District Lahore and Kasur of Punjab, Pakistan

**DOI:** 10.3390/pathogens10050620

**Published:** 2021-05-18

**Authors:** Shahzad Ali, Usama Saeed, Muhammad Rizwan, Laiba Hassan, Muhammad Ali Syed, Falk Melzer, Hosny El-Adawy, Heinrich Neubauer

**Affiliations:** 1 Wildlife Epidemiology and Molecular Microbiology Laboratory (One Health Research Group), Discipline of Zoology, Department of Wildlife & Ecology, Ravi Campus, University of Veterinary and Animal Sciences, Lahore, Pattoki 54000, Pakistan; usama.saeed@uvas.edu.pk (U.S.); rizwanasif400@gmail.com (M.R.); 2 Department of Microbiology, The University of Haripur, Haripur 22620, Pakistan; laibahassan1996@gmail.com (L.H.); mirwah2000@yahoo.de (M.A.S.); 3 Institute of Bacterial Infections and Zoonoses, Friedrich-Loeffler-Institut, Naumburger Str. 96a, 07743 Jena, Germany; falk.melzer@fli.de (F.M.); Hosny.ElAdawy@fli.de (H.E.-A.); heinrich.neubauer@fli.de (H.N.); 4 Faculty Medicine of Veterinary, Kafrelsheikh University, Kafr El-Sheikh 33516, Egypt

**Keywords:** brucellosis, occupational hazard, molecular detection, risk factors

## Abstract

Brucellosis is a neglected zoonotic disease, and occupations with a high risk of infection exist. Limited information is available on brucellosis for these employees at high risk in Pakistan. A total of 459 persons with high-risk occupations, i.e., 211 abattoir workers, 63 milkers, 52 dung cake makers, 44 veterinarians, 44 shepherds, and 45 veterinary students of the districts Kasur and Lahore, Pakistan, were tested in this study. Blood samples and information on place of residence, gender, age, urbanicity, type of occupation, socioeconomic status, contact with animals, consumption of raw milk, contact to women who had a miscarriage, contact to aborted animal fetus or abortion material, pregnancy, miscarriage history, or intrauterine fetal death were collected. Serum samples were examined using Rose Bengal tests for anti-*Brucella* antibodies and seropositive samples were subjected to genus-specific qPCR for the detection of DNA. Data were analyzed using chi-squared and binary regression. Twenty (4.35%) persons were seropositive for anti-*Brucella* antibodies. Out of these, 18 (90%) were tested positive by *Brucella* genus-specific qPCR. Positive sera were more often found in Lahore district (8.3%) than in Kasur district (3.1%). Persons older than 36 years were more often seropositive. Persons involved in the handing of milk and in contact with animals were more often seropositive. Contact with women who had had a miscarriage was also a significant factor for seropositivity for brucellosis. Place of residence, gender, age, urbanicity, and contact with animals were identified as potential risk factors in the present study. The present study confirmed that brucellosis is an occupation hazard for abattoir workers, milkers, dung cake makers, and veterinarians in the districts of Lahore and Kasur of Punjab, Pakistan. The study shows the need for strategies for safety at work to minimize the risk of infection. Raising awareness for the prevention and use of proper personal protection equipment during the slaughtering and treatment of animal is highly needed.

## 1. Introduction

Brucellosis is a major public health problem caused by the bacterial genus *Brucella* (*B.*). It is an important zoonosis of farm animals, i.e., cattle, pigs, goats, or sheep. According to the World Health Organization (WHO), about five hundred thousand new human cases are reported annually [1]. It has been eradicated in most developed countries, but it is still endemic in some countries in Africa, the Mediterranean region, parts of Asia, and the Americas [2]. The genus *Brucella* contains eleven species, each one with individual host preferences, epidemiology, and pathogenicity. *B. abortus* infects cattle, *B. melitensis* infects goats and sheep, *B. suis* infects pigs, *B. canis* infects dogs, *B. ovis* infects sheep, *B. neotomae* infects dessert wood rats, *B. micoti* infects common voles [3], *B. ceti* infects cetaceans, and *B. pinnipedialis* infects seals [4]. *B. inopinata* was isolated from a human breast implant infection [5]. Recently, two species of this genus were isolated from baboons and red foxes, named *B. papionis* and *B. vulpis,* respectively [6,7].

Brucellae are Gram-negative, intracellular bacteria. Animal brucellosis is characterized by late abortion, orchitis, arthritis, and becomes chronic in most cases. Human brucellosis has become a serious threat to public health, although it remains neglected. Currently, *B. abortus*, *B. melitensis,* and *B. suis* are reported as the main causative agents of brucellosis in humans. In some rare human cases, *B. canis* has also been described as the causative agent [8]. Brucellosis is definitely a disease of animals, because humans are dead-end hosts [9,10,11].

The role of wildlife in the spread of cross-species brucellosis infection is unknown. *Brucella* strains, e.g., *B. suis*, have been isolated from wild animals such as wild boars, and there is need to analyze the risk of transmission of these *Brucella* strains for livestock and human health [12,13,14,15]. Transmission occurs via the inhalation of aerosolized bacteria, entry through skin abrasions or intact skin, or especially the ingestion of raw dairy products (raw milk, raw meat, etc.), because brucellae are excreted with abortion material, lochia, or milk in high numbers. Occupationally exposed groups such as livestock farmers, milkers, veterinary students, abattoir workers, shepherds, and laboratory workers are at high risk of infection [16,17].

Prevalence and incidence of disease might vary among countries. Brucellosis is a contagious systemic disease with a variety of clinical symptoms. Clinical diagnosis is difficult because it has symptoms similar to malaria or fever of unknown origin [1]. Symptoms such as weakness, body pain, septicemia, or undulant fever characterize the acute stage. Later on, the disease may become chronic, and sequelae such as arthritis are prominent [18,19]. Various methods are used to diagnose human brucellosis, i.e., cultivation, serological methods, and PCR assays. Cultivation is time-consuming and hazardous to personnel, therefore serological methods are preferred tests to diagnose human brucellosis. In developing countries, the Rose Bengal plate test is used as a first test because it is easy to perform, reliable, and inexpensive [20].

In rural areas of Pakistan, 16% of the human population in close contact to animals was reported as seropositive for brucellosis [21]. High-risk professionals (6.9%), and pregnant women (8.5%) were recorded to be infected with brucellosis [9,22]. The need for a study to assess the risk for different groups of personnel involved in dairy food production, i.e., livestock farmers, veterinarians, livestock farm workers, and milkers became obvious. Thus, this study was planned to identify the risk factors related with brucellosis for high-risk professionals in two districts of Punjab, Pakistan.

## 2. Results

A total of 459 human blood samples were collected in Kasur and Lahore (Table 1). Twenty (4.35%) samples were found to be RBPT-positive. Eighteen (90%) of these samples were also positive in *Brucella* genus-specific qPCR. The seroprevalence was higher in Lahore (8.3%) than in Kasur (3.1%), a finding which was statistically significant (*p* ≤ 0.05). The prevalence of anti-*Brucella* antibodies varied from one tehsil to another. The location was found to be a risk factor. It was highest in Model town tehsil (15.4%), and lowest in Pattoki (1.8%) (Table 1).

Gender was found to be a potential risk factor (*p* ≤ 0.05) by binary logistic regression, where men were more often found to be seropositive (5.4%) than women (1.6%). Age group was also found to be a potential risk factor. Six (6.2%) and eight (6.2%) participants younger than 50 and younger than 40 were RBPT-positive, respectively. Fewer participants (1.6%) of the ≤20 age group and ≤30 age group (3.8%) were RBPT-positive (Table 2 and Table 3).

A higher RBPT prevalence was found in urban areas (5.4%) than in rural areas (3.8%). This finding was statistically insignificant (*p* ≥ 0.05). Seroprevalence values of veterinary students, shepherds, veterinary professionals, dung cake makers, milkers, and abattoir workers were 0%, 0%, 2.3%, 1.9%, 7.9% and 6.2%, respectively. The latter were at higher risk of infection with brucellosis, which was proven by binary logistic regression (*p* ≤ 0.006) (Table 3).

In the present study, the socioeconomic status was identified as a significant risk factor for brucellosis (*p* ≤ 0.05), because participants of low economic status were more often seropositive than (4.5%) middle class individuals (4.1%). Of the 411 individuals who reported contact with animals, 12 (2.9%) were seropositive, whereas 8 (16.7%) of the 48 individuals reporting no contact with animals were seropositive. Contact was found to be a potential risk factor for brucellosis (*p* ≤ 0.05) (Table 3).

Of the 264 individuals who confirmed that they drank raw milk, 12 (4.5%) were seropositive, whereas only 8 (4.1%) out of the 195 participants who said that they never drink raw milk were found seropositive. Sixty-nine participants had contact with women who had aborted: 6 (8.7%) were seropositive vs. 14 (3.6%) of the 390 participants who reported no contact. Contact with women who had had a miscarriage was found to be a significant factor for brucellosis (*p* ≤ 0.05). The study showed that contact with an aborted animal fetus was an insignificant risk factor (*p* ≥ 0.05), proven by binary logistic regression. A total of 151 persons had had contact with such fetuses, and 7 (4.6%) were seropositive, while 13 (4.2%) of the 308 participants without contact were positive. None of the 30 pregnant women with one pregnancy were found to be seropositive. Sixty-eight pregnant women with two pregnancies participated in the study, and two (2.9%) were seropositive. No seropositive case was found in those 11 women who had had more than three pregnancies. None of the 16 women who had never been pregnant were RBPT positive. One (1.7%) of the 58 women with miscarriage history and one (1.5%) of the 67 women without miscarriage history were seropositive (*p* ≥ 0.05). One of the 75 women who reported intrauterine fetal death was seropositive, and one (2%) of the 50 women without complications was also found to be positive.

## 3. Discussion

Brucellosis is a neglected bacterial disease with a high risk of human infections. It causes significant economic damage and public health problems, especially in developing countries such as Pakistan [18]. Brucellosis is transmitted to humans mainly through contact with infected animals or the consumption of contaminated raw animal products [23]. Brucellosis causes non-specific symptoms in humans and sequelae are likely to occur. Diagnosis still relies on serology for the confirmation of brucellosis [24], because culture is hazardous for personnel.

The present study was conducted to determine the prevalence and risk factors related with brucellosis in high-risk occupational groups from the districts of Lahore and Kasur. This study reported the overall prevalence to be 4.35% in 459 samples. The prevalence in groups that were comparable to this group but from neighboring countries, India and Bangladesh, were reported to be 1.8% and 2.0%, respectively [25,26]. However, a comparatively high seroprevalence of 21.7% was observed in a previous study conducted in slaughterhouse workers of Lahore [27]. A possible reason for that finding is the difference in the compilation of the sampling groups of both studies. All participants of the previous study were slaughterhouse workers (animal keeper, loader, vet/paravet, slaughterer, meat seller, cleaner, etc.) who had maximum exposure to many different animal species. Additionally, the consumption of raw milk did put them at high risk of acquiring foodborne zoonoses such as brucellosis. Our statement about the transmission of brucellosis from animals to humans is supported by the detection of brucellosis in dairy animals of districts Kasur and Lahore in previous studies [10,11].

The seroprevalence was higher in men (5.4%) than in women (1.6%). This finding can be explained by the risk groups themselves: the occupational groups of this study are dominated by men (i.e., veterinarians, shepherds, milkers, abattoir workers). Similarly, studies from neighboring countries, i.e., Bangladesh and India also reported a higher seroprevalence in men (5.6% and 12.24%, respectively) as compared to women (0.8% and 0.0%, respectively), because the involvement of men in livestock management in these countries is traditionally high [28,29]. Comparatively high seroprevalences in male workers was also reported in Iran (13.3%), Portugal (11.1%), and China (34%) [30,31,32]. These data on variation are in accordance with those of a previous study conducted in the Potohar region of Pakistan [9].

This study reported the highest seroprevalence in participants older than 36 years of age, followed by the age groups of 26–35 years and younger than 26 years. It can be supposed that prolonged exposure without taking protective measures results in higher prevalence. Higher age was also found to be associated with a higher seroprevalence in Bangladesh [28]. In contrast to this study, research conducted in Saudi Arabia and India reported seroprevalences to be higher in study participants younger than 40 years [33,34]. In Potohar region, Pakistan, the seroprevalence (11.3%) was high in participants younger than 30 years because most of these study participants again were slaughterhouse workers [9].

This study recorded a higher prevalence in people from urban areas (5.4%) than from rural areas (3.8%). The obvious reason was that persons tested in the present study had occupations more common in urban than in rural people. This study was contrary to other studies in which prevalence was found to be high in rural areas. Brucellosis prevalence was reportedly three times higher in rural (21.4%) than in urban areas (7.9%) in Uganda [35]. In Iran, prevalence in rural areas (1.7%) was comparable to that in urban areas (1.0%) [36]. In general, persons from rural areas are more often involved in dairy farming and livestock management, i.e., in milking and handling of cow dung, and are thus at higher risk of exposure.

The highest seroprevalence among occupational groups was found in milkers (7.9%), followed by abattoir workers (6.2%), and then other occupations such as veterinarians, dung cake makers, shepherds, and veterinary students. Milkers and abattoir workers might have been unaware of the risk and did not follow precautionary measures during milking and butchering. Milkers, especially, seem to be at a high risk of infection with brucellosis, or at least to develop anti-*Brucella* antibodies. In Bangladesh, milkers had a prevalence of 18.2%, in contrast to veterinary practitioners (5.3%), butchers (2.5%), and livestock farmers (2.6%) [28].

The seroprevalence of people of low socioeconomic status (4.5%) was not significantly higher than that of people of middle socioeconomic status (4.1%). Although poor families did not seek medical treatment due to financial constraints, unawareness of the family doctors may have led to failure to provide adequate medical treatment, resulting in frequent high prevalence. Furthermore, significant prevalence of brucellosis is reported from low-income countries [37]. Most important risk factors for transmission were contact with women who had had miscarriages (8.7%), contact with aborted animal fetus (4.6%), consumption of raw milk (4.5%), and direct contact with animals (2.9%). Many previous studies have reported that direct contact with animals is indeed one of the most important risk factors for brucellosis.

Aborted fetuses of animals contain large numbers of *Brucella* organisms, therefore they must be properly disposed of in order to prevent further transmission of brucellosis. Likelihood of infection increased significantly in abattoir workers involved in the handling of aborted fetuses in Nigeria [38]. Similar findings regarding the association of human brucellosis cases with the handling of placentas and aborted fetuses of infected animals were also reported from Chad and Tanzania [18,39,40]. The risk factor “contact to persons with miscarriage” reflects either a common problem of public health in Pakistan or indeed a problem of poverty and ignorance. This problem is alarming and needs immediate clarification for the sake of mothers and their unborn children. Consumption of raw milk from infected animals mainly contributed to disease acquisition in the study participants. A study conducted in Uganda reported high prevalence in those who kept livestock (17.1%) and consumed unpasteurized milk (14.7%) as well [41]. Awareness for the zoonotic risk of food needs to increase in Pakistani consumers, especially those living in urban areas.

Pregnancy and the number of pregnancies were recorded in women. The highest seroprevalence was found in women who had had more than two pregnancies, while no seropositive case was found in women who had had no, one and more than two pregnancies. The miscarriage history (1.7%) recorded in women of the present study was found to be the lowest when compared to other studies. The reasons might be that most of these pregnant women had taken proper precautions to avoid brucellosis during pregnancy. A study conducted in Iran in pregnant women reported the history of spontaneous abortion to be 19.9% [42]. Another study conducted by Elshamy and Ahmed in Saudi Arabia recorded a 27.2% spontaneous abortion rate in seropositive pregnant women, showing a relationship between brucellosis and abortion [43].

One of the complications of brucellosis during pregnancy is intrauterine fetal death. About 1.3% of pregnant women with anti-*Brucella* antibodies reported intrauterine fetal death. This is similar to the numbers in a study conducted in Saudi Arabia, which recorded 2% of intrauterine fetal deaths in 92 *Brucella*-positive pregnant women in the third trimester of pregnancy [44]. A fairly high proportion of intrauterine fetal deaths of 3.45% was recorded in 29 pregnant women with brucellosis in a tertiary care hospital in Turkey [45]. Special awareness programs for family doctors and gynecologists could help to start well-timed medication.

*Brucella* DNA was detected in samples of 18 out of 20 seropositive persons. This number is comparable to the number of positive results in samples of high-risk professionals reported in the Potohar region of Pakistan [9]. PCR-based detection and confirmation of *Brucella* infection in human and animal samples is the contemporary state-of-the-art diagnosis [12,28,46,47].

## 4. Materials and Methods

### 4.1. Sampling Site

**Figure 1:**
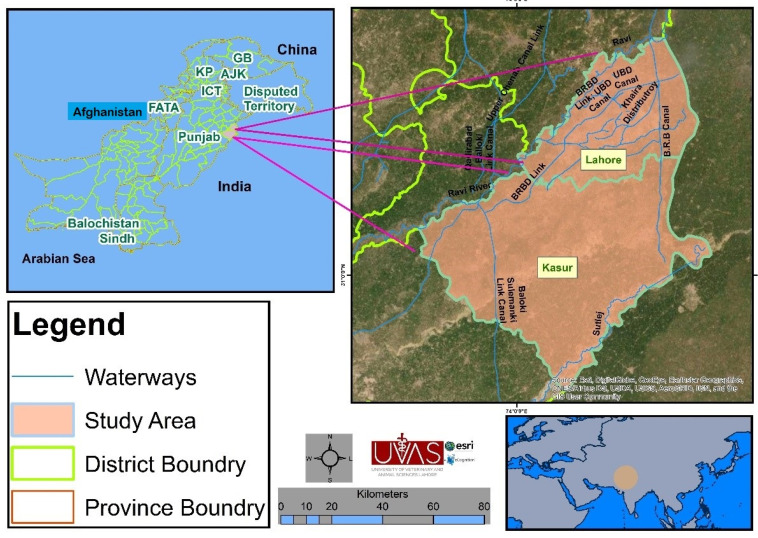
Map of the study area which consists of two districts (Lahore and Kasur).

The present study was conducted in two districts (Kasur (31.0896° N; 74.1240° E) and Lahore (31.4313° N; 74.3587° E)) of Punjab Province, Pakistan (Figure 1). Both districts are located adjacent to each other in central zones of Punjab. Kasur district is divided in four tehsils (an administrative sub-division of a district), including Kasur, Pattoki, Chunian, and Kot Radha Kishan. Kasur city is the capital of Kasur district. Kasur district stretches over 3995 km^2^. It has borders with the districts of Lahore, Faisalabad, and Nankana Sahib, and India to the east, west, north, and south, respectively. The total human population is 3,454,996 in the district of Kasur, where 25.78% of the human population live in urban and 74.22% live in rural areas. Most of the human population lives in rural areas of Kasur district and are actively involved in livestock (i.e., cows (88,321 households), buffaloes (175,298 households), sheep (16,990 households), goats (94,337 households) and camels (919 households)) rearing. Lahore district is divided in five tehsils, including Model town, Lahore city, Lahore cantt., Shalimar, and Raiwind. Lahore is a densely populated city and the capital of the province of Punjab, Pakistan. It stretches over 1772 km^2^. The total human population is more than eleven million; 82.44% of the population live in urban areas, and 17.56% live in rural areas [48]. It has borders with India (Wagah border) to the east, Kasur district to the south, the River Ravi is to the north, while Sheikhupura district is situated on its north and west side. Most of the people of peri-urban and rural areas are engaged in livestock (i.e., cows (40,153 households), buffaloes (64,997 households), sheep (19,253 households), goats (48,983 households) and camels (1908 households)) rearing. Livestock is important for the income of the rural population and contributes to the economy in terms of wool, milk, meat, fat, bone, hide, and blood production [49,50].

### 4.2. Data Collection and Informed Consent

A questionnaire was used to collect the basic data for descriptive epidemiology, i.e., district, tehsil, gender, urbanicity, age, occupation, contact with animals, socio-economic status, raw milk consumption, contact with women who had had a miscarriage, miscarriage history, and intrauterine fetal death. Informed consent was obtained from all participants. The questionnaire was first developed in the English language and translated to the local languages for better understanding of the questions by the participants.

### 4.3. Sample Collection

A total of 459 blood samples were randomly collected from high-risk professionals, including veterinary students, shepherds (owners of large and small ruminants), vets and their assistants, dung cake makers, milkers (milk collectors from farms), and abattoir workers (blood collectors, leather cleaners, knackers, butchers, etc.). Blood (3 mL) was collected in anticoagulant free tubes, placed in an ice box, and immediately transferred to the laboratory for further analysis. Then, samples were centrifuged at 5000 rpm for 4 min for the extraction of serum. The supernatants were collected in 1.5 mL labelled tubes and kept at −20 °C until investigation.

### 4.4. Serology

Serum samples were screened for the presence of anti-*Brucella* antibodies using RBPT antigen (ID Vet, France). Thirty microliters each of antigen and serum were spotted on glass plates, mixed gently, and agitated for 4 min to observe agglutination. Any agglutination was considered positive. Positive and negative serum samples were used as controls.

### 4.5. Molecular Detection of Brucella DNA

Extraction of DNA from positive sera was conducted by WizPrep gDNA Mini Kit (Wizbio solutions Inc., Seongnam, Korea) according to the manufacturer’s instructions. qPCR was performed on a MJ Mini Bio-RAD Thermal cycler (Applied Biosystems, Foster City, CA, USA). Genus-specific primers and probes targeting the BCSP-31 gene were used [51]. qPCR was performed and results were evaluated as described in a previous study [52].

### 4.6. Statistical Analysis

A chi-squared test was used to analyze the association between risk factors and test outcomes in the Statistical Package for Social Sciences (SPSS 21.0). To calculate the confidence interval (CI) and to assess the prevalence, a binomial logistic regression was run. To check the prevalence of human brucellosis and check the relationship between variables, odd ratios along with 95% CIs were calculated. A *p*-value ≤ 0.05 was considered significant [10].

## 5. Conclusions

This study reports that individuals with occupations exposing them to farm animals and their products are at a high risk of acquiring brucellosis in an area where farm animal brucellosis is endemic. The surveillance of human brucellosis is essential to monitor the success of countermeasures. High-risk occupational groups must be made aware of zoonotic diseases, and they must be educated on precautionary measures to protect themselves and to reduce the risk for consumers as well. There is a need for periodically screening high-risk personnel for zoonotic diseases (i.e., brucellosis) in endemic areas to identify persons who should receive immediate and specific treatment.

## Figures and Tables

**Table 1:** District and tehsil-wise prevalence of brucellosis in high-risk professionals of Pakistan.

Variable		Category	Examined	Infected	Prevalence (%)	Chi-Squared	*p*-Value
**District**		Kasur	350	11	3.1	5.216	0.027
		Lahore	109	9	8.3		
**Tehsils**	Lahoreregion	Model town	39	6	15.4	16.223	0.006
		Lahore city	45	2	4.4		
		Lahore cantt.	25	1	4		
	Kasurregion	Chunian	85	4	4.7		
		Pattoki	227	4	1.8		
		Kasur	38	3	7.9		

**Table 2:** Factors associated with seropositivity of brucellosis in high-risk professionals from Pakistan.

Variable	Category	Examined	Infected	Prevalence (%)	Chi-Squared	*p*-Value
**Gender**	Male	334	18	5.4	3.134	0.057
	Female	125	2	1.6		
**Age**	18–25	129	2	1.6	4.335	0.04
	26–35	104	4	3.8		
	36–40	129	8	6.2		
	>40	97	6	6.2		
**Urbanicity**	Rural	292	11	3.8	0.671	0.413
	Urban	167	9	5.4		
**Occupation**	Veterinary students	45	0	0	8.837	0.03
	Shepherds	44	0	0		
	Veterinarians	44	1	2.3		
	Dung cake makers	52	1	1.9		
	Milkers	63	5	7.9		
	Abattoir workers	211	13	6.2		
**Socioeconomic status**	Low	242	11	4.5	0.043	0.835
	Medium	217	9	4.1		
**Contact with animals**	Yes	411	12	2.9	19.490	0
	No	48	8	16.7		
**Consumption of raw milk**	Yes	264	12	4.5	0.053	0.818
	No	195	8	4.1		
**Contact with aborted women**	Yes	69	6	8.7	3.668	0.05
	No	390	14	3.6		
**Contact with aborted fetus**	Yes	151	7	4.6	0.042	0.838
	No	308	13	4.2		
**No of pregnancies**	1	30	0	0	1.704	0.737
	2	68	2	2.9		
	3	11	0	0		
	No	16	0	0		
**Miscarriage history**	Yes	58	1	1.7	0.011	0.918
	No	67	1	1.5		
**Intrauterine fetal death**	Yes	75	1	1.3	0.085	0.711
	No	50	1	2		

**Table 3:** Results of the logistic regression analysis, odds ratio with 95% confidence interval, and two-sided *p*-value for binary logistic regression.

Variable	95% CI		Odds Ratio	*p*-Value
	Lower	Upper		
District	0.001	0.111	0.011	0
Tehsil	0.658	2.695	1.331	0.426
Gender	3.243	977	56.291	0.006
Age	0.622	2.428	1.299	0.04
Urbanicity	2.956	113.6	18.328	0.002
Occupation	0.403	1.286	0.720	0.006
Socio economic status	0.281	4.588	1.136	0.858
Contact with animals	0.000	0.028	0.002	0
Consumption of raw milk	0.382	7.003	1.635	0.508
Contact with aborted person	2.686	152.838	20.263	0.004
Contact with aborted fetus	0.079	3.016	0.487	0.439
No. of pregnancies	0.902	3.267	1.717	0.100
Miscarriage history	0.490	25.284	3.519	0.211
Intra uterine fetal death	0.046	3.531	0.403	0.411

## Data Availability

The data presented in this study are available within the article.
